# Molecular Basis of LH Action on Breast Cancer Cell Migration and Invasion via Kinase and Scaffold Proteins

**DOI:** 10.3389/fcell.2020.630147

**Published:** 2021-02-05

**Authors:** Joselina Magali Mondaca, Ivonne Denise Uzair, Ana Carla Castro Guijarro, Marina Inés Flamini, Angel Matias Sanchez

**Affiliations:** ^1^Laboratorio de Transducción de Señales y Movimiento Celular, Instituto de Medicina y Biología Experimental de Cuyo (IMBECU), Consejo Nacional de Investigaciones Científicas y Técnicas (CONICET), Universidad Nacional de Cuyo, Mendoza, Argentina; ^2^Laboratorio de Biología Tumoral, Instituto de Medicina y Biología Experimental de Cuyo (IMBECU), Consejo Nacional de Investigaciones Científicas y Técnicas (CONICET), Universidad Nacional de Cuyo, Mendoza, Argentina

**Keywords:** LH, cortactin, N-WASP, migration and invasion, breast cancer

## Abstract

Breast cancer (BC) is a major public health problem affecting women worldwide. Approximately 80% of diagnosed cases are hormone-dependent breast cancers. These hormones are known to stimulate tumor development and progression. In this setting, tentative evidence suggests that luteinizing hormone (LH) may also play a role in tumors. In BC cells that express functional LH receptors (LHR), this hormone regulates cell migration and invasion by controlling several kinases that activate actin cytoskeletal proteins. In this article, we show that LH induces phosphorylation of paxillin and its translocation toward the plasmatic membrane, where focal adhesion complexes are assembled. This process is triggered via a rapid extra-gonadal LHR signaling to Src/FAK/paxillin, which results in the phosphorylation/activation of the nucleation promoter factors cortactin and N-WASP. As a consequence, Arp2/3 complexes induce actin polymerization, essential to promote cell adhesion, migration, and invasion, thus enhancing metastatic spread of tumoral cells. Our findings provide relevant information about how gonadotrophins exert their action in BC. This information helps us understand the extragonadal effects of LH on BC metastasis. It may provide new perspectives for therapeutic treatment, especially for women with high serum levels of gonadotrophins.

## Introduction

Women produce a range of reproductive hormones. Their secretion patterns vary throughout their lifetime. In premenopausal women, hormone levels fluctuate due to physiological variations associated with the menstrual cycle. Postmenopausal women have lower estrogen and higher gonadotrophin concentrations, but in general, their hormone levels are fairly constant (Hall et al., 2000; Hernandez et al., 2005). Gonadotrophins, such as follicle-stimulating hormone (FSH) and luteinizing hormone (LH), are glycoproteins that act on the gonads to regulate development and reproduction. These hormones are produced by the anterior pituitary gland. Their secretion is induced by the gonadotrophin-releasing hormone (GnRH), which is released in a pulsatile manner in response to circulating levels of estrogens and progesterone via a negative feedback (Tsutsumi and Webster, 2009).

The role of LH in the female gonads is well established. It exerts its action by binding to specific LH receptors (LHR) that are part of the heptahelical G-protein-coupled receptor family (Ascoli et al., 2002). Thus, LH influences the production of androgens, which are aromatized to estrogens. After ovulation, gonadotrophins act to maintain progesterone levels, controlling steroidogenesis and gametogenesis (Gharib et al., 1990).

Although ovaries are the classic and single questionless target for gonadotrophins, recent research has demonstrated that receptors for this hormone are also present in normal and tumoral extragonadal tissue, including in the breast (Meduri et al., 1997, 2003; Jiang et al., 2002; Rao and Lei, 2007; Sanchez et al., 2016). In fact, LHR is widely expressed in breast tumors (Meduri et al., 1997, 2003) and BC cell lines (Bodek et al., 2003; Sanchez et al., 2016, 2018). Significantly higher LH levels and LHR expression have been found in invasive tumors, suggesting that LH upregulation could play a key role in breast carcinogenesis (Silva et al., 2002; Hudelist et al., 2009). We recently reported that LH modulates the expression of a set of genes implicated in tumorigenesis in BC cells and that the circulating levels of gonadotrophins are directly correlated with tumor growth in an *in vivo* model of BC (Sanchez et al., 2018). For all these reasons, and in accordance with findings of other groups, we suggest that an LH-triggered mechanism of action exists that could affect the development and/or progression of BC (Tanaka et al., 2000; Meduri et al., 2003; Hudelist et al., 2009; Sanchez et al., 2016, 2018).

The worst prognoses of BC are closely linked with the ability of tumors to generate metastasis at distant sites. Around 90% of deaths in BC patients are due to the development of metastasis (Redig and McAllister, 2013). This process occurs through specific steps that result in alterations in the adhesion, migration, and invasion properties of tumor cells, ultimately triggering metastatic spread (Jiang et al., 2015).

A dynamic reorganization of the actin cytoskeleton is key to the metastatic process. It is modulated by the action of several fundamental kinases and scaffold proteins, such as Src and focal adhesion kinase (FAK) (McLean et al., 2005). In our previous work, we identified that LH/LHR stimulates BC cell migration and invasion via a rapid signal to Gαi/Gβ in an Src and FAK-dependent pathway (Sanchez et al., 2016). Phosphorylated FAK recruits and activates paxillin, a scaffold protein that acts as a docking site for many actin cytoskeletal regulators (Shortrede et al., 2016).

Cortactin is a scaffold protein involved in branching of actin filaments (Uzair et al., 2019). When cortactin is activated, it relays signals from Src/FAK-paxillin to the Arp2/3 complex, leading to actin nucleation (Kruchten et al., 2008). N-WASP belongs to the family of the Wiskott-Aldrich syndrome proteins (WASPs). It acts as a scaffolding protein, recruiting signals from cdc42 GTPases for their regulation. Cortactin and N-WASP synergistically control the Arp2/3 complex (Uruno et al., 2001), enhancing the formation of actin-based protrusive structures involved in cell migration and invasion (Frugtniet et al., 2015).

Although elevated LH levels have been associated with a worse prognosis (Pujol et al., 2001), knowledge about the molecular mechanism by which LH exerts its action in BC remains poor. The aim of the present article was therefore to further our understanding of the molecular signaling induced by LH on BC cell morphology and motility. In particular, we were interested in investigating the influence of LH on the migratory, invasive, and metastatic potential of BC cells.

## Materials and Methods

### Cell Culture and Treatments

The T-47D human breast carcinoma cell line was obtained from the American Type Culture Collection (ATCC, United States). T-47D cells were grown in RPMI 1640 supplemented with L-glutamine (2 mM), 10% fetal bovine serum (FBS), penicillin, and streptomycin under 5% CO_2_ atmosphere at 37°C. Prior to the experiments investigating non-transcriptional effects, BCs were kept in a medium containing no FBS for 8 h. LH (Luveris 75 IU) was obtained from the Merck Serono Laboratory. The concentration was chosen to mimic follicular phase levels (5 mIU/ml), since it induces a greater phosphorylation/activation on key regulatory proteins of cell motility (Sanchez et al., 2016). Different chemical inhibitors were used: 4-amino-5-(4-chlorophenyl)-7-(*t*-butyl)-pyrazolo-(3,4-d) pyrimidine (PP2, 10 μM) was from Calbiochem, and FAK inhibitor (FAKi, 1 μM), Wiskostatin (10 μM), and CK-666 (4 μM) were from Santa Cruz Biotechnology. Whenever an inhibitor was used, the compound was added 45–60 min before starting the active treatments. PP2, FAKi, Wiskostatin, and CK-666 were dissolved in DMSO.

### Immunoblottings

Cell lysates were separated by SDS-PAGE in 8–10% gels and transferred into polyvinylidene difluoride (PVDF) membranes. Antibodies used were p-FAK^Y397^ (611807), FAK (610088), and Arp3 (612135) (BD Transduction Laboratories); actin (sc-1615), LHR (sc-25828), c-Src (sc-5266), paxillin (sc-31010), p-paxillin^Y118^ (sc-365020), cortactin (sc-11408), p-cortactin^Y466^ (sc-101611), N-WASP (sc-13139), Arp2 (sc-15389), and p-Tyr (sc-7020) (Santa Cruz Biotechnology); p-N-WASP^*S*484/485^ (ab1964) (Chemicon International); p-Src^Y418^ (ab4816) (Abcam); and p-Arp2^*T*237^ (orb155730) (Biorbyt). Primary and secondary antibodies were incubated with the membranes using standard techniques. Immunodetection was accomplished using enhanced chemiluminescence and recorded with a quantitative digital imaging system (ChemiDoc XRS with Image Lab, Bio-Rad).

### Cell Immunofluorescence

T-47D cells were grown on coverslips and exposed to different treatments. Cells were fixed with 4% paraformaldehyde for 30 min and permeabilized with 0.1% Triton for 5 min. Blocking was performed with phosphate-buffered saline (PBS) containing 3% bovine serum albumin (BSA) for 30 min at room temperature. Cells were incubated with antibodies against p-N-WASP^S484/485^ (Chemicon International) and p-paxillin^Y118^ and p-cortactin^Y466^ (Santa Cruz Biotechnology) overnight at 4°C, followed by incubation with DyLight 594 and/or fluorescein-conjugated secondary antibody (FITC 1:150; Vector Laboratories). Cells were then incubated with Texas Red–phalloidin (Sigma-Aldrich) for 30 min. After washing, the nuclei were counterstained with 4’-6-diamidino-2-phenylindole (DAPI) (Sigma-Aldrich) and mounted with VECTASHIELD mounting medium (Vector Laboratories, Burlingame, CA). Immunofluorescence was visualized using a Nikon Eclipse E200 microscope and recorded with a high-resolution DP70 Olympus digital camera.

### Co-immunoprecipitation Assay

T-47D cells were harvested with ice-cold lysis buffer containing 20 mM Tris–HCl pH 7.4, 10 mM EDTA, 100 mM NaCl, IGEPAL 0.5%, 3 μl/ml protein inhibitor cocktail (Sigma Aldrich, P8340), 3.3 μl/ml phosphatase inhibitor cocktail (Sigma Aldrich, P0044), 0.1 mg/ml phenylmethylsulfonyl fluoride (PMSF), and 3 mM sodium orthovanadate. In lysis buffer, 500 μg/μl of protein were mixed with 2 μg of FAK, cortactin, or Arp2 primary antibody and incubated for 1 h at 4°C with gentle rocking. Then, 40 μl 1:1 of protein-A agarose (sc-2001, Santa Cruz Biotechnology) was added. The mixture was gently rocked for 2 h at 4°C and centrifuged at 12,000×*g* for 5 min at 4°C. The supernatant was removed, and the immunoprecipitates washed with 500 ml of 20 mM Tris–HCl pH 7.4, 10 mM EDTA, 150 mM NaCl, 1% IGEPAL, 1 mM Na_3_VO_4_, 50 mM NaF, 0.1 mg/l PMSF, 0.3 mg/l aprotinin, and 0.01% protease inhibitor mixture (Sigma-Aldrich). Immunoprecipitated proteins were separated under reducing and denaturing conditions by 10% SDS-PAGE and transferred to a PVDF membrane. Non-specific binding was blocked with 3% BSA in PBS–Tween 20. Membranes were incubated with anti-FAK, anti-cortactin, anti-Arp2, anti-Arp3, and p-Tyr antibodies.

### Gene Silencing With RNA Interference

**FIGURE 1:**
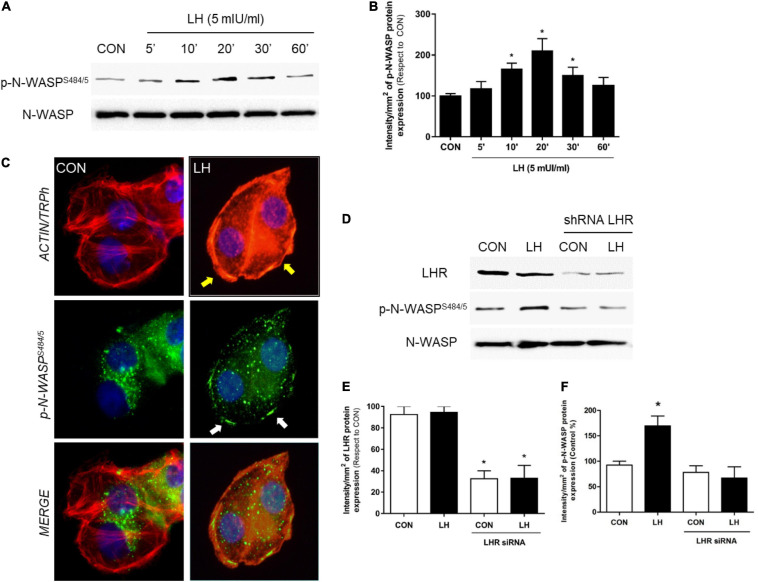
LH signals to LHR, enhancing N-WASP phosphorylation. **(A)** T-47D cells were treated for different times (0–60 min) with LH (5 mIU/ml). The total cell amounts of wild-type N-WASP and phosphorylated N-WASP (p-N-WASP^S484/5^) are shown by western blot analysis. **(B)** Phospho-N-WASP densitometry values were adjusted to N-WASP intensity and then normalized to the control sample. *P < 0.05 vs. control. **(C)** T-47D BC cells were treated with LH (5 mIU/ml) for 20 min. Cells were stained with phospho-N-WASP^S484/5^ linked to fluorescein isothiocyanate (FITC, green), actin fibers with Texas Red phalloidin (red), and nuclei were counterstained with DAPI. Yellow arrows indicate actinic cytoskeleton reorganization to the periphery of the cell membrane. White arrows indicate membrane-localized p-N-WASP. **(D)** T-47D BC cells were transfected with shRNA vs. LHR or with vehicle, and protein analyses for LHR, N-WASP, and phospho-N-WASP^S484/855^ were performed on cell lysates with or without treatment for 20 min with LH. The total cell amounts of wild-type LHR, N-WASP, and phospho-N-WASP^S484/485^ are shown by western blot. **(E,F)** LHR and phospho-N-WASP densitometry values were adjusted to N-WASP intensity and then normalized to the control sample. The results are expressed as the mean ± *SD* of the measurements. *P < 0.05 vs. CON, control. All experiments were performed in triplicate; representative images are shown.

**FIGURE 2:**
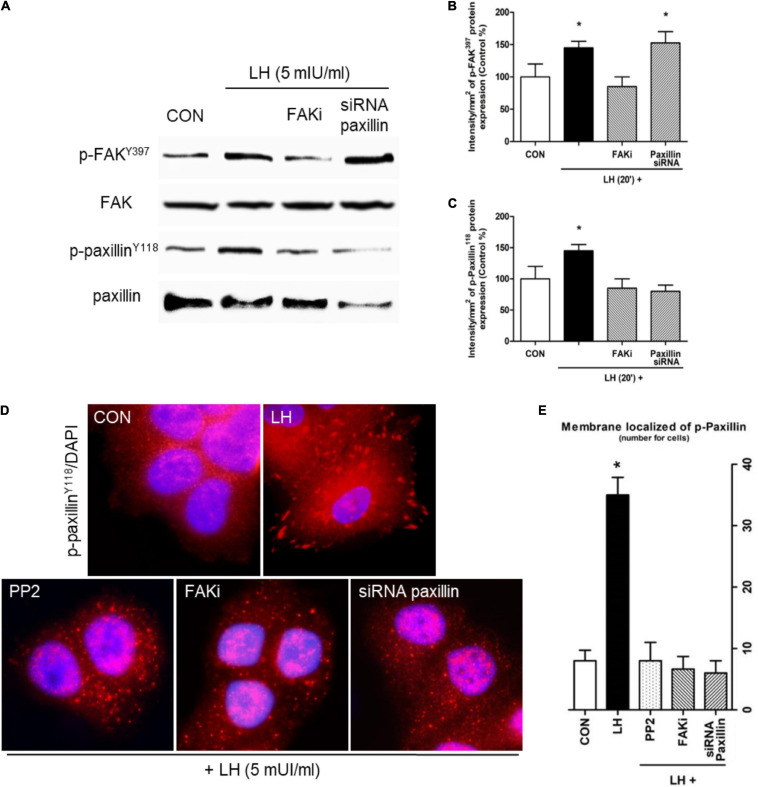
LH promotes FAK and paxillin phosphorylation through LHR. **(A)** Cells were treated with LH (5 mIU/ml) for 20 min in the presence or absence of FAKi (1 μM) and siRNA paxillin. The total cell amounts of wild-type FAK and paxillin, phospho-FAK^Y397^, and p-paxillin^Y118^ are shown by western blot. **(B,C)** Phospho-FAK^Y397^ and phospho-paxillin^Y118^ densitometry values were adjusted to FAK and paxillin intensity, respectively, and then normalized to the control sample. Results are expressed as the mean ± *SD*. *P < 0.05 vs. control. **(D)** BC cells were stained vs. phospho-paxillin^Y118^ linked to DyLight 594 and nuclei counterstained with DAPI. **(E)** Quantification of the membrane-localized p-paxillin^Y118^ in the different conditions. Results are expressed as dots/cells (mean ± SD). *P < 0.05 vs. CON, control. Membrane-localized p-paxillin^Y118^ was counted in 40 different cells. All experiments were performed in triplicate, and representative images are shown.

Synthetic small interfering RNAs targeting paxillin (siRNA paxillin), Cdc42 (siRNA Cdc42), and control siRNAs were purchased from Santa Cruz Biotechnology. SureSilencing shRNA Plasmid Human LHCGR (Cat KH01310G) and control shRNA were purchased from SuperArray Bioscience Corporation. The siRNAs were used at a final concentration of 50 nM using Lipofectamine 2000 (Invitrogen). T-47D BC cells were treated 48 h after siRNA transfection. The efficacy of gene silencing was checked with western blot analysis and found to be optimal at 48 h. Control experiments demonstrating selectivity and efficacy of silencing of the different targets can be found in Figures 1D, 2A.

### Transfection Experiments

A dominant negative construct for cortactin (cortactin^3YF^, a non-phosphorylated mutant of cortactin) was generously donated by Dr. John Cooper (Washington University School of Medicine, United States). The inserts were cloned in pcDNA 2AB Flag-cortactin 3YF (Tehrani et al., 2007). A mutant construct for N-WASP (N-WASP^Δ^ ^VCA^, N-WASP truncation mutant ΔVCA domain) that lacks the carboxy-terminal domain necessary to activate Arp2/3 complex (Kovacs et al., 2011) was provided by Dr. Alpha Yap (University of Queensland, Australia). The plasmids (10 μg) were transfected into T-47D cells using Lipofectamine 2000 (Invitrogen, United States). BC cells were treated 24–48 h after transfection. The efficacy of transfection was checked with western blot analysis and found to be optimal at 36 h.

### Adhesion Assay

Five hundred thousand cells per well were seeded into six-well plates on coverslips previously coated with 1% sterile gelatin and exposed to different treatments. The cells were incubated at 37°C for 2 h. Non-adherent T-47D cells were then removed by gentle washing with PBS. The attached cells were fixed with 4% paraformaldehyde and stained with 10% ethanol/crystal violet for 20 min. Cells of attached images were captured and counted in 10 randomly chosen fields per well using a Nikon Eclipse E200 microscope coupled to a high-resolution CCD digital camera, as previously described (Flamini et al., 2014).

### Cell Migration Assay (Wound-Healing Assay)

Cell migration was assayed with razor scrape assays. Briefly, a razor blade was pressed through the confluent T-47D BC cell monolayer into the plastic plate to mark the starting line. T-47D cells were swept away on one side of that line. Cells were washed, and 2.0 ml of RPMI 1640 containing steroid-deprived FBS and gelatin (1 mg/ml) was added. To prevent cell proliferation, cytosine β-D-arabinofuranoside hydrochloride (Sigma) (10 μM), a selective inhibitor of DNA synthesis that does not inhibit RNA synthesis, was used 1 h before the test substance was added. Migration was monitored for 48 h. Cells were digitally imaged, and the migration distance was measured using a Nikon Eclipse E200 microscope and recorded with a high-resolution DP70 Olympus digital camera. Percentage of migration was calculated and reported as a percentage of the control.

### Cell Invasion Assay

Cell invasion was assayed using the BD BioCoat^TM^ Growth Factor Reduced (GFR) Matrigel^TM^ Invasion Chamber (BD Bioscience, United States). In brief, after rehydration of the GFR Matrigel inserts, the test substance was added to the wells. An equal number of control inserts (no GFR Matrigel coating) were prepared as control. Added into the inserts was 0.5 ml of T-47D cell suspension (2.5 × 10^4^ cells/ml). Cytosine β-D-arabinofuranoside hydrochloride (Sigma) (10 μM), a selective inhibitor of DNA synthesis that does not inhibit RNA synthesis, was used 1 h before the test substance was added to prevent cell proliferation. The chambers were incubated for 48 h at 37°C, 5% CO_2_ atmosphere. After incubation, the non-invading cells were removed from the upper surface of the membrane using cotton-tipped swabs. The cells on the lower surface of the membrane were then stained with Diff-Quick stain. The invading cells were observed and photographed under the microscope at 100 × magnification. Cells were counted in the central field of triplicate membranes.

### Statistical Analysis

Statistical analysis of the data was performed with one-way analysis of variance (ANOVA) followed by Tukey–Kramer multiple-comparisons test using GraphPad Prism 5.03 software. *P* < 0.05 was considered statistically significant. All values are expressed as mean ± *SD* of three independent experiments.

## Results

### LH Triggers N-WASP Phosphorylation Through LH Receptor

N-WASP is essential in the regulation of actin nucleation, leading to changes in cell morphology and consequently stimulating cell migration, invasion, and metastasis. We have previously reported that N-WASP is phosphorylated after 17β-estradiol and triiodothyronine treatment (Sanchez et al., 2010; Shortrede et al., 2016; Uzair et al., 2019), increasing cell membrane structure formation implicated in cellular movement. Therefore, as a first approach to establish the extragonadal actions of LH on BC cell motility, T-47D cells were treated with follicular-phase levels of LH (5 mIU/ml) for different times (0–60 min) to analyze the expression and phosphorylation of N-WASP, a key regulator of actin cytoskeleton reorganization (Frugtniet et al., 2015). We found that LH promoted a rapid increase of N-WASP phosphorylation on Ser^484/485^ in a time-dependent and transient manner. This effect was highest after 20 min and returned to basal levels after 60 min (Figures 1A,B).

In parallel, we performed an immunofluorescence assay to evaluate the cellular location of N-WASP after LH treatment (5 mIU/ml, 20 min). Phosphorylated N-WASP^S484/485^ was homogeneously distributed throughout the cytoplasm in the control cells. In cells exposed to LH, a rapid actinic cytoskeleton reorganization from the cytoplasm to the periphery of the cell membrane occurred (Figure 1C, yellow arrows). Also, p-N-WASP^S484/485^ translocated to the edge of the membrane where it co-localized with actin fibers (Figure 1C, white arrows), promoting a thickening of the membrane (Figure 1C, black arrows) and inducing actinic nucleation.

In order to determine whether LHR is involved in the control of N-WASP, we silenced LHR with a specific shRNA. This resulted in a significant reduction of LHR expression along with a dramatic decrease in p-N-WASP^S484/485^ after LH treatment (Figures 1D–F), thus confirming the role of LHR in N-WASP phosphorylation.

### LH Induces a Dynamic Actin Cytoskeletal Reorganization via Src/FAK/Paxillin in BC Cells

Focal adhesion complexes (FA) undergo changes that ultimately lead to metastatic spread (Sanchez et al., 2010). We therefore analyzed proteins involved in FA, such as Src, FAK, and paxillin, in BC cells. We observed that a rapid pulse of LH for 20 min increases FAK^Y397^ and paxillin^Y118^ phosphorylation (Figures 2A–C). The presence of a specific FAKi reduced both FAK^Y397^ and paxillin^Y118^ phosphorylation, while the use of siRNA paxillin decreased paxillin^Y118^ phosphorylation (Figures 2A–C). These results suggest the existence of a signaling pathway involving FAK and paxillin in the regulatory mechanism of LH on BC cells.

We used immunofluorescence to evaluate the subcellular localization of p-paxillin^Y118^ in T-47D cells. We observed that LH increased paxillin^Y118^ phosphorylation and translocation from the cytoplasm to the membrane, generating FA (Figures 2D–E). Treatment with Src inhibitor (PP2), FAKi, and siRNA paxillin impaired these LH-induced events.

### LH Signals to Cortactin Through a Paxillin-Dependent Signaling Pathway

**FIGURE 3:**
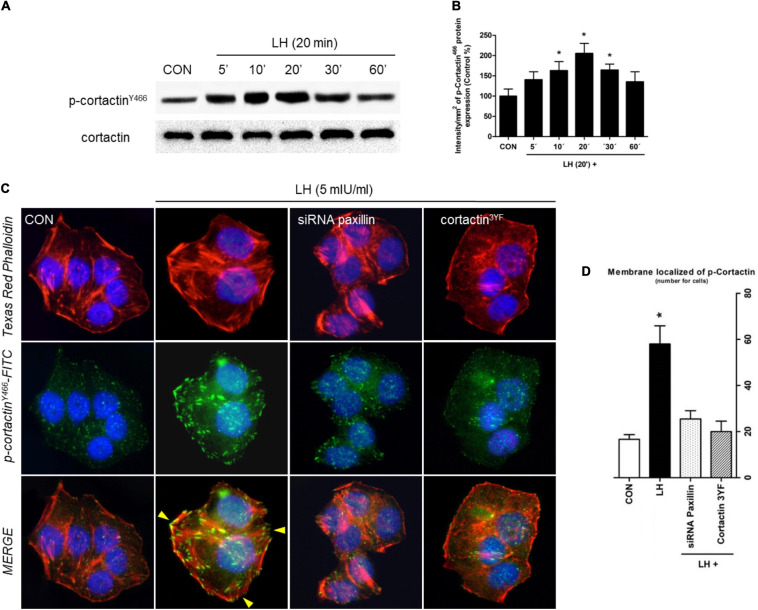
LH induces dynamic actin remodeling in BC cells. **(A)** BC cells were treated for different times (0–60 min) with LH (5 mIU/ml). The total cell amounts of wild-type cortactin and phosphorylated cortactin (cortactin^Y466^) are shown by western blot analysis. **(B)** Phospho-cortactin^Y466^ densitometry values were adjusted to cortactin intensity and then normalized to the control sample. *P < 0.05 vs. control. **(C)** T-47D cells were treated with LH for 20 min in the presence or absence of a siRNA paxillin or cortactin^3YF^ construct. Cells were stained with phospho-cortactin^Y466^ linked to FITC, actin was stained with phalloidin linked to Texas Red, and nuclei were counterstained with DAPI. Yellow arrows indicate membrane-localized p-cortactin^Y466^. **(D)** Quantification of the membrane-localized p-cortactin in the different conditions. Results are expressed as dots/cells (mean ± *SD*). *P < 0.05 vs. CON, control. Membrane-localized p-cortactin^Y466^ was counted in 40 different cells. All experiments were performed in triplicate, and representative images are shown.

Since cortactin is another key regulator of cell motility, migration, and invasion, we determined its phosphorylation in T-47D cells exposed to 5 mIU/ml of LH for different times (0–60 min) (Figures 3A,B). Maximal cortactin^Y466^ phosphorylation was found at 20 min; it receded to basal levels after 60 min. A similar pattern of phosphorylation was found with N-WASP (Figures 1A,B).

To test whether LH induces actin cytoskeleton reorganization via cortactin, we performed an immunofluorescence assay. Cells treated with LH for 20 min triggered cortactin^Y466^ phosphorylation and translocation to FA, where it co-localized with the thickening membrane (Figures 3C,D, yellow arrows). Treatment with siRNA paxillin and a dominant negative construct for cortactin (cortactin^3YF^) impaired phosphorylation and the consequent translocation of p-cortactin^Y466^ to the plasma membrane (Figures 3C,D). These results suggest that LH signals to cortactin via paxillin.

### LH Controls the Arp2/3 Complex via the FAK/Paxillin/Cortactin/N-WASP Cascade

**FIGURE 4:**
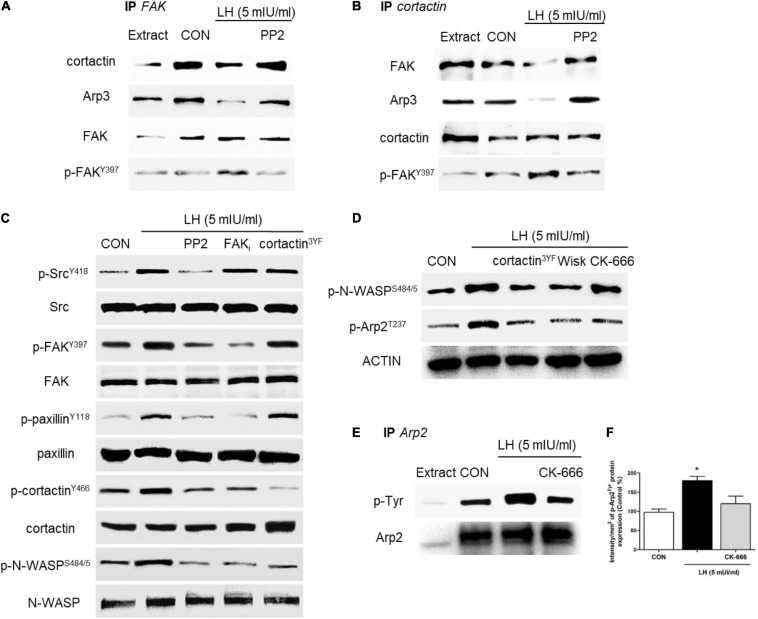
LH stimulates the Src/FAK/paxillin/cortactin/N-WASP and Arp2/3 complexes. **(A,B)** BC cells were exposed to LH (5 mIU/ml) for 20 min in the presence or absence of the c-Src kinase inhibitor PP2 (10 μM). Cell protein extracts were immunoprecipitated with an antibody vs. FAK **(A)** and cortactin **(B)**. The immunoprecipitates (IPs) were assayed for co-immunoprecipitation vs. FAK, p-FAK, cortactin, and the Arp3 subunit. The membranes were re-blotted for the immunoprecipitated protein to show equal input. **(C)** Cells were incubated in the presence of LH (5 mIU/ml) for 20 min with or without PP2 (10 μM), FAK (1 μM), and/or dominant negative constructs vs. cortactin (cortactin^3YF^). The total cell amounts of wild-type c-Src, FAK, paxillin, cortactin, and N-WASP or p-Src^Y416^, p-FAK^Y397^, p-paxillin^Y118^, p-cortactin^Y466^, and p-N-WASP^*S484/485*^, respectively, are shown by western blot. **(D)** BC cells were transfected with the dominant negative constructs of cortactin (cortactin^3YF^), the specific inhibitor of N-WASP (Wiskostatin, 10 μM), and/or the inhibitor of the Arp2/3 complex (CK-666, 4 μM) and incubated in the presence of LH (5 mIU/ml) for 20 min. Phospho-N-WASP^*S484/485*^ and phospho-Arp2^*T*237^ were assayed by western blot analysis. **(E,F)** T-47D cells were exposed to LH (5 mIU/ml, 20 min) in the presence or absence of CK-666. Cell protein extracts were immunoprecipitated (IP) with an antibody vs. Arp2. IP was assayed for co-immunoprecipitation vs. p-Tyr. The membranes were re-blotted for the immunoprecipitated protein vs. Arp2 to show equal input. All experiments were performed in triplicate with consistent results; representative images are shown. *P < 0.05 vs. CON, control.

The Arp2/3 complex is central to the rapid actin network formation toward the periphery of the cell membrane, which is required to build cellular structures for cell motility, such as filopodia and lamellipodia. This complex is activated by two regulator proteins, cortactin and N-WASP, which act alone or synergistically (Helgeson et al., 2014) to promote actin branching and enhance BC cell migration and invasion (Uzair et al., 2019). To continue identifying the signaling pathway triggered by LH on BC cells, we assessed the role of FAK, cortactin, N-WASP, and the Arp2/3 complex, which are the main regulators of FA and actin nucleation (Uzair et al., 2019). We performed two co-immunoprecipitation assays (IP) in BC cells treated with LH (5 mIU/ml, 20 min) in the presence or absence of PP2. We found that the basal interaction between FAK/cortactin and FAK/Arp3 was significantly reduced when cells were treated with LH. This effect was impaired by the use of the specific inhibitor PP2 (Figure 4A). In addition, we observed that LH reduced the interaction of cortactin/FAK and cortactin/Arp3 compared to control, but PP2 treatment blocked this reduction (Figure 4B). Our results suggest that, in basal condition, the FAK/cortactin/Arp3 subunit interacts in BC cells. After LH treatment, this interaction was partially dissociated, leading to FAK phosphorylation in Tyr^397^ via the Src kinase (Figures 4A,B). FAK phosphorylation in Tyr^397^ is fundamental for a conformational change that allows FAK protein to expose the Tyr^397/407/576/577/861/925^ residues for autophosphorylation (McLean et al., 2005; Sanchez et al., 2010).

In parallel, we tested the role of several kinases and scaffold proteins involved in the signaling to the Arp2/3 complex. T-47D cells treated with LH increased Src^Y419^, FAK^Y397^, paxillin^Y118^, cortactin^Y466^, and N-WASP^S484/485^ phosphorylation, and these increments were prevented by the use of PP2. Blockade with FAKi resulted in a visible inhibition of FAK^Y397^, paxillin^Y118^, cortactin^Y466^, and N-WASP^S484/485^ phosphorylation, whereas transfection with cortactin^3YF^ prevented phosphorylation of cortactin^Y466^ and N-WASP^S484/5^ (Figure 4C).

We also assessed whether phosphorylation of cortactin and N-WASP may regulate the Arp2/3 complex after LH stimulation. We found that LH significantly increased N-WASP^S484/5^ and Arp2^*T*237^ phosphorylation. Transfection with cortactin^3YF^ and the specific inhibitor for N-WASP (Wiskostatin) resulted in a reduction of N-WASP^S484/5^ and Arp2^*T*237^ phosphorylation. The use of the Arp2/3 complex specific inhibitor (CK-666) also prevented Arp2^*T*237^ phosphorylation (Figure 4D). Furthermore, we demonstrated that Arp2 is activated after LH treatment, as shown by the increase of phospho-tyrosine in Arp2 immunoprecipitates (Figures 4E,F). This effect was abolished by CK-666. All these findings suggest a signaling cascade involving LHR, Src, FAK, paxillin, cortactin, and N-WASP that mediates the regulatory effects of LH on the Arp2/3 complex in BC cells.

### LH Effect on BC Cell Adhesion, Migration, and Invasion

**FIGURE 5:**
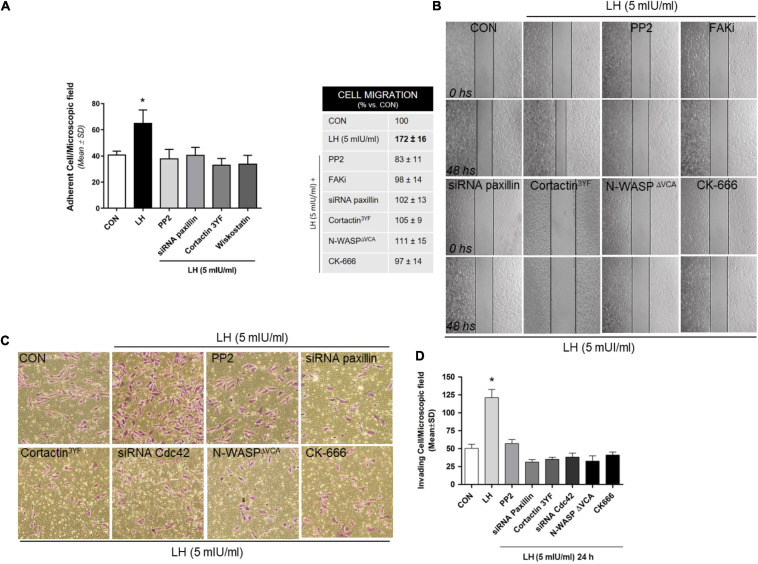
LH modulates cell adhesion, migration, and invasion in BC cells. **(A)** Cells were treated with LH (5 mIU/ml) for 20 min in the presence or absence of the specific inhibitor PP2, Wiskostatin, or CK-666 and transfected with siRNAs vs. paxillin or mutant constructs for cortactin (cortactin^3YF^). Numbers indicate quantity of attached cells per field. Experiments were performed in triplicate; *P > 0.05 vs. control. **(B)** Cells were treated with LH (5 mIU/ml) for 48 h in the presence or absence of PP2, FAKi, and CK-666. Other cells were transfected with siRNAs toward a paxillin, cortactin^3YF^, and N-WASP^Δ*VCA*^ construct. Representative images are shown. The upper black lines indicate the starting line, and the lower black lines the mean migration distance. Cell migration gap closure was quantified by the use of the NIH ImageJ software. Values are presented as a percentage of control. *P < 0.05 vs. control. The experiments were performed in triplicate. **(C,D)** T-47D cells were treated with LH (5 mIU/ml) in the presence or absence of different inhibitors or siRNAs, as indicated in **(A,B)**, and siRNA Cdc42. BC cell invasion through Matrigel was assayed with invasion chambers. Representative images in chambers with Matrigel are shown. Invading cells were counted in the central field, and the graph indicates the mean number of invading cells ± *SD* from three separate experiments. *P < 0.05 vs. CON, control.

Metastasis occurs through specific steps that result in alterations in the adhesion, migration, and invasion properties of tumor cells (Jiang et al., 2015). Thus, to relate the molecular action of LH on BC cell motility, we performed cell adhesion, migration, and invasion assays. Treatment with LH significantly enhanced the ability of T-47D cells to adhere to a gelatin matrix in comparison with the control cells (Figure 5A). The adhesion capacity of the cells was diminished when they were exposed to specific inhibitors or silencers, such as PP2, siRNA paxillin, cortactin^3YF^ and Wiskostatin compared to LH treatment alone (Figure 5A).

We then performed a wound-healing assay to evaluate cell migration in T-47D cells exposed to LH (5 mIU/ml). Treatment with LH significantly increased BC cell migration after 48 h of exposition compared to control cells (Figure 5B). Blockade of Src, FAK, paxillin, cortactin, N-WASP, and the Arp2/3 complex reduced the migration process, demonstrating the involvement of these proteins in LH-induced cell migration.

To determine the capacity of BC cells to invade the surrounding environment, we performed three-dimensional invasion assays using Matrigel. LH-treated cells showed an enhanced invasion after 24 h compared with control cells (Figures 5C,D). Inactivation of Src, paxillin, cortactin, Cdc42, N-WASP, and the Arp2/3 complex suppressed LH-induced BC cell invasion (Figures 5 C,D).

## Discussion

Breast cancer develops in a hormonal environment that determines tumor behavior and influences clinical response to therapy (Redig and McAllister, 2013). Most BCs express estrogen receptors, indicating estrogen dependence of the tumor (Tokunaga et al., 2014), and are treated with therapies that reduce estrogen levels or inhibit their receptors. Since gonadotrophins and their receptors are involved in estradiol synthesis, alterations in their signaling could modify estrogen levels and, consequently, influence BC progression. Some studies have reported that LH and LHR genes may suffer mutations that alter estrogen levels. Hence, exposure to LH may increase BC risk (Haavisto et al., 1995; Powell et al., 2003). Gonadotrophins have been proposed as modulators in BC development, but the available evidence is limited and inconsistent (Mann et al., 2003; Ziecik et al., 2007; Huhtaniemi, 2010).

The key findings of this work are that LH regulates the cell adhesion, migration, and invasion processes via the phosphorylation of fundamental actin cytoskeletal proteins in T-47D BC cells. As we recently reported, this process is promoted by the recruitment of functional LH receptors that are expressed in these cells (Sanchez et al., 2016, 2018). LH, via LHR, triggers the recruitment of several kinase and scaffold proteins through a non-genomic pathway, leading to an increased phosphorylation and translocation of N-WASP and promoting BC movement. N-WASP is a key nucleation promoter factor; its deregulation has been involved in the invasion, intravasation, and metastasis of mammary tumors (Frugtniet et al., 2015). We have recently described that N-WASP promotes cell migration and invasion of BC cells after being activated by several hormones, such as triiodothyronine (Uzair et al., 2019) and estradiol (Sanchez et al., 2010; Shortrede et al., 2016). Our previous findings highlight the importance of N-WASP’s actin branching ability, which could thus be considered a potential therapeutic target for invasion and metastasis inhibition in diverse types of cancers, including BC. Indeed, Hebbrecht et al. (2017) developed nanobodies that target the N-WASP^*VCA*^ domain responsible for Arp2/3 activation, thus causing a decrease in invadopodia formation in BC cells (Hebbrecht et al., 2017). This approach could lead to the development of a novel anticancer drug that limits the metastatic potential of cancer cells.

We also evaluated the influence of LH on FA regulation. In T-47D cells, LH rapidly increases FAK and paxillin phosphorylation with the consequent translocation to FA, enhancing the formation of specialized cell membrane structures involved in cellular motility. We have previously reported that several hormones exert a regulatory effect on FA activation. The formation of FA is one of the first steps to impulse actin cytoskeleton reorganization and accomplish molecular motility (Sanchez et al., 2010; Shortrede et al., 2016; Uzair et al., 2019). Paxillin is a crucial component of FA. Once phosphorylated, it serves as a scaffolding molecule that mediates FA assembly and turnover (Lopez-Colome et al., 2017). Furthermore, it plays an important role in neoplastic transformation due to its ability to directly bind to several oncogene proteins, such as Src and FAK kinases, disrupting normal adhesion and growth factor signaling cascades necessary to impulse migration and invasion (Shortrede et al., 2016; Lopez-Colome et al., 2017). Several studies have reported that paxillin overexpression is associated with alterations and malignant progression of breast tumors (Madan et al., 2006; Short et al., 2007). Thus, paxillin may be used as a prognostic biomarker. It could, potentially, also have implications for therapeutic approaches targeted at preventing invasion.

We next explored the role of cortactin, another nucleation-promoting factor (NPF) that regulates the activation of the Arp2/3 complex either alone or synergistically with N-WASP (Takenawa and Suetsugu, 2007; MacGrath and Koleske, 2012). We determined that LH increases cortactin^Y466^ phosphorylation and translocation to FA in a Src/FAK/paxillin-dependent manner. Blockage of this signaling with specific inhibitors, such as siRNAs or mutant constructs, significantly reduces cell adhesion, migration, and invasion, which reveals the fundamental role of actin nucleation proteins in tumorigenesis. In recent years, great progress has been made in understanding the role of cortactin and its molecular mechanism in cell motility. Cortactin has been considered as an invadopodial marker, as the ability of cancer cells to form invadopodia is often correlated with their invasive and metastatic capabilities (Meirson and Gil-Henn, 2018). miRNAs targeting the cortactin gene have been shown to inhibit invadopodial formation in human lung cancer (Li et al., 2018), whereas its overexpression enhances cell migration in oral cancer (Ramos-Garcia et al., 2019). Treatment with specific inhibitors of NPF could thus be an interesting approach to counteract metastasis. Dasatinib, a drug that disrupts the Src/cortactin signaling, is currently being tested as a therapeutic to block the action of NPF (Meirson and Gil-Henn, 2018).

Cortactin is an actin-binding and adaptor-scaffolding protein with binding sites for diverse target proteins, including Src, FAK, and the Arp3 subunit. It acts as a central molecule between FA formation and actin nucleation, which are key steps in the regulation of cell motility (MacGrath and Koleske, 2012; Tomar et al., 2012). Here, we demonstrate that LH disrupts the basal FAK/cortactin/Arp3 subunit interaction and that this effect can be prevented with the specific Src inhibitor (PP2). Similarly, we recently reported that sex steroid treatment diminishes the association between cortactin and the Arp3 subunit in cortical neuron cells (Uzair et al., 2020). We propose that the specific phosphorylation of these proteins affects their interaction as a consequence of physical impediments involving a cycle of binding, phosphorylation, and subsequent dissociation accompanied by FA turnover and cell movement.

Our results evidence that a tight regulation of Arp2/3 is crucial for cell migration, invasion, and metastasis. Several chemical inhibitors of the Arp2/3 complex, such as CK-666, are currently available; they arrest cell motility by impairing actin branching. Further research on inhibitors of Arp2/3 is needed to better understand the mechanisms of Arp2/3 activity, as its uncontrolled activation is correlated with the onset and progression of many diseases, including BC (Chanez-Paredes et al., 2019).

Gonadotrophins are an important component of the menopausal transition. During the latter, LH and FSH serum levels increase significantly over a period of 3–9 years (Landgren et al., 2004). This variation may lead to physiological changes that impact women’s health. Diverse pathologies seem to be associated with elevated gonadotrophin levels. LH has previously been described as a cell enhancing migration and invasion by activating regulator proteins in ovarian (Mertens-Walker et al., 2010), endometrial (Noci et al., 2008), and breast cancer (Sanchez et al., 2016). Casadesus et al. (2007) described that increased LH levels are associated with declines in cognitive performance. Human chorionic gonadotrophin (hCG), which shares the α-subunit with LH and acts through the same LHR receptor (Choi and Smitz, 2014), increases gastric cancer cell proliferation through the PKA/c-Met signaling pathway (Zhao et al., 2018). Regarding LHR, its overexpression in endometrial cancer cells increases invasiveness, tumor development, and distal metastasis (Pillozzi et al., 2013). All this evidence highlights the diverse extragonadal actions of gonadotrophins and their physiological consequences.

Altogether, our experiments evidence the rapid signaling of LH through extragonadal LHR to the Src/FAK/paxillin/cortactin–N-WASP/Arp2/3 complex, enhancing BC cell adhesion, migration, and invasion. Our results highlight that LH could promote BC progression, particularly in postmenopausal women in which the absence of a menstrual cycle leads to an increase in circulating levels of gonadotrophins. Hence, a potential therapeutic approach in BC patients could be to regulate gonadotrophin levels.

There are widely available drugs that reduce the synthesis and release of LH/FSH via GnRH agonists and antagonists. GnRH agonists suppress sex steroid levels and are used as an adjuvant treatment of hormone-sensitive tumors, such as prostate or breast cancer (Chengalvala et al., 2003; Huhtaniemi et al., 2009). Prostate cancer patients are treated with LHR agonists as a first-line therapy to downregulate LHR expression in the pituitary gland, which leads to a reduced androgen synthesis (Liu et al., 2010). The same approach has been proposed to treat patients with LHR + urinary bladder invasive cancer (Szepeshazi et al., 2012). Some studies, however, suggest that patients treated with gonadotrophins to induce ovulation (Pappo et al., 2008) or with drugs that increase their circulating levels (Lerner-Geva et al., 2006; Orgeas et al., 2009) may have a higher BC risk. Assuming that gonadotrophins might promote BC, it would be primordial in postmenopausal women who have higher circulating levels of LH and FSH and the highest BC incidence (Bray et al., 2004).

**FIGURE 6:**
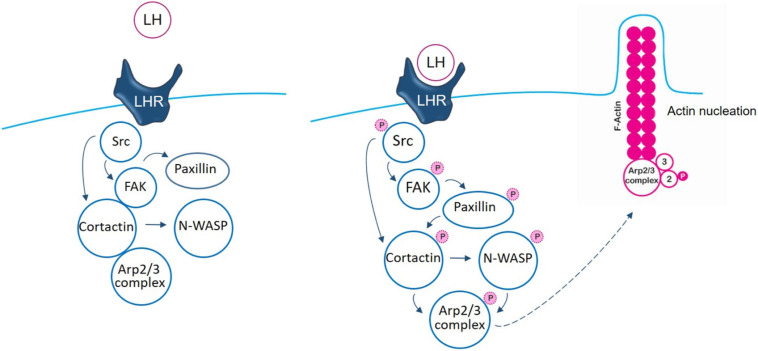
Schematic signaling cascade triggered by LH, promoting BC cell adhesion, migration, and invasion. In control T-47D BC cells, there is an interaction between FAK/cortactin/Arp3 subunit. Binding of LH to LHR led to FAK/cortactin/Arp3 disruption, inducing FAK-Tyr^397^ phosphorylation, via Src kinase. This phosphorylation induces a conformational change that allows FAK protein its complete autophosphorylation. When FAK is phosphorylated, it signals to paxillin to finally modulate the Arp2/3 complex, via cortactin and N-WASP, enhancing actin nucleation and promoting BC cell motility.

The initial steps induced by LH to Src/FAK kinases, via LHR, have been previously reported by our group (Sanchez et al., 2016). The main contribution of this manuscript is that we reveal important new aspects based on the analysis of nuclear promoter factors, cortactin and N-WASP, to the control of the actin regulator Arp2/3 complex which participates in the actin nucleation process. We have continued elucidating the signaling pathway where LH triggers, via LHR, the phosphorylation of Src/FAK to the paxillin/cortactin–N-WASP/Arp2/3 complex, controlling BC cell adhesion, migration, and invasion (schematic Figure 6). Understanding the molecular mechanisms impulsed by LH in BC pathology is key for the development of original clinical strategies or new drugs that decrease the metastatic potential of gonadotrophin-sensitive cancers.

## Data Availability Statement

The original contributions presented in the study are included in the article/supplementary material, further inquiries can be directed to the corresponding author/s.

## Author Contributions

JM carried out different experiments, cell culture, and treatments. IU performed immunofluorescence and migration assays. AC performed co-immunoprecipitation and invasion assays. MF was instrumental in funding the study and participated to the writing of the manuscript. AS planned and funded the project, supervised the experiments, wrote the manuscript. All authors contributed to the article and approved the submitted version.

## Conflict of Interest

The authors declare that the research was conducted in the absence of any commercial or financial relationships that could be construed as a potential conflict of interest.
